# 

**Published:** 1904-12

**Authors:** 


### CONTENTS.

ORIGINAL COMMUNICATIONS—

LUMBAR PUNCTURE, A DIRECT METHOD FOR THE TREATMENT OF TETANUS WITH THE REPORT OF NINE SUCCESSFUL CASES SO TREATED. By W. Troy Bivings, A B., PH.B , M.D., Atlanta, Ga ....... 577

COMPULSORY RETURNS OF ALL CASES OF PULMONARY TUBERCULOSIS THE FIRST STEP TOWARDS PUBLIC MEASURES OF PREVENTION AND TREATMENT. By Eugene R. Corson, M.D., Savannah, Ga. ....... 586

ACUTE ALCOHOLISM. By C. C. Stockard, M.D., Atlanta, Ga ....... 591

A CASE OF CALCULUS. By W. L. Champion, M.D., Atlanta, Ga ....... 594

EDITORIAL NOTES AND COMMENTS—

AN EIGHTEENTH CENTURY TUBERCULOSIS CONCEIT ....... 596

GEORGIA STATE COMMISSION ON TUBERCULOSIS ....... 599

REORGANIZATION AND THE A. M A. ....... 601

NEWS AND NOTES ....... 603

CONTENTS CONTINUED ....... X

The
Grand Prize
awarded to
MELLIN'S
FOOD
over all other infants' foods
THE HIGHEST AWARD
of the
World's Fair Saint Louis
This is even higher than the Gold Medal

Please mention this Journal.

No physician can afford to be indifferent regarding the accurate filling of his prescriptions.

SELECTIONS AND ABSTRACTS—

Some Points in the Diagnosis and Treatment of Pneumonia.................. 606
Proprietaries.................................................. 613
Advice to Young Doctors; Sick-Room Manners........................... 617
A Preliminary Fifth Year in the Medical Curriculum........................ 621
Dietary of the Child in Health and Disease.............................. 624
Autointoxication.................................................. 626
Medical Education in Japan......................................... 630
A Few Practical Points in Infantile Anemia.............................. 632
Written Instructions to Patients.................................... 634
Will Cure Bite of Most Deadly Snakes.................................. 635
Indoor Humidity.................................................. 637
How May the Public School be Helpful in the Prevention of Tuberculosis...... 638
Castor Oil and Epsom Salts made Palatable.............................. 639
The Trained Nurse................................................ 640
The Telephone as an Alcoholometer..................................... 641
A Few Remarks on Asthma........................................... 642
The Status of Mechanical Vibration in Therapeutics........................ 643
Hot and Cold Water in Eye Diseases.................................... 644
Bronchopneumonia................................................ 644
Damiana Used at Home............................................. 645
Vaseline in Constipation............................................ 646
A Perfected Food................................................. 646
Thrush.......................................................... 647
The Sewing-Machine and Health....................................... 647
Urticaria on Arms and Chest......................................... 648
Elixir in Bronchial Irritation, Coryza and Catarrh........................ 648
An Antiseptic Vaginal Tampon........................................ 648

MEDICAL ITEMS—Continued..............................xvi, xviii, xx, xxii, xxiv

